# The miR-146a Single Nucleotide Polymorphism rs2910164 Promotes Proliferation, Chemoresistance, Migration, Invasion, and Apoptosis Suppression in Breast Cancer Cells

**DOI:** 10.3390/cells14080612

**Published:** 2025-04-18

**Authors:** Sarai Morales-González, Gloria M. Calaf, Mónica Acuña, Julio C. Tapia, Lilian Jara

**Affiliations:** 1 Núcleo Interdisciplinario de Biología y Genética, Instituto de Ciencias Biomédicas (ICBM), Facultad de Medicina, Universidad de Chile, Independencia, Santiago 8380000, Chile; sarai.morales@ug.uchile.cl (S.M.-G.); macuna@med.uchile.cl (M.A.); 2 Instituto de Alta Investigación, Universidad de Tarapacá, Arica 1010069, Chile; gmcalaf@academicos.uta.cl

**Keywords:** breast cancer, microRNA, SNP, proliferation, migration, invasion, apoptosis

## Abstract

Breast cancer (BC) is the most common malignant disease in women worldwide. Several studies have reported that microRNA-146a (miR-146a) dysregulation plays a role in multiple cancers, including BC. However, the mechanism underlying this association is controversial, possibly reflecting diverse roles for this miR in different types of cancer. The SNP rs2910164:G>C, located within the miR-146a precursor, has been linked to a BC risk. Our group previously showed a specific association between rs2910164:G>C and an increased BC risk in patients with early-onset sporadic BC. There are no studies in the literature that evaluate the functional consequences of the rs2910164 polymorphism in the BC process. Therefore, the goal of the present study was to evaluate in vitro the effect of the SNP rs2910164:G>C on BC progression in luminal A and triple-negative cell lines. We found that rs2910164:G>C upregulated the expression of two mature miR-146a sequences, 3p and 5p. Furthermore, pre-miR-146a-C enhanced proliferation, migration, and invasion in luminal A and triple-negative breast cells, as well as decreasing cisplatin-induced apoptosis. Interestingly, the pre-miR-146a C allele decreased cisplatin resistance in MCF-7 cells but increased cisplatin resistance in MDA-MB-231 cells. We propose that the rs2910164 C allele promotes miR-146a overexpression, which is causally involved in proliferation, migration, invasion, apoptosis, and cisplatin resistance.

## 1. Introduction

Currently, breast cancer (BC) is the most prevalent cancer in women worldwide, and one in eight women will develop BC during their lifetimes [1,2]. In Chile, BC mortality in women suffered an important incremental increase over four decades, being 8.5/100,000 in 1985 and 18.2/100,000 in 2022 [3,4]. Genetic factors play a key role in BC etiology, in which mutations found in the *BRCA1* and *BRCA2* genes have been of the most studied; however, they only account for about 16% of familial cases [5].

A large amount of evidence has emerged to support the role of microRNAs (miRNAs) in BC development and progression. MiRNAs are a class of endogenous, noncoding, single-stranded RNAs of 20–25 nucleotides of extension. It is well known that miRNAs regulate expression by either promoting the degradation or blocking the translation of its mRNA targets by mainly binding to their 3′-UTR [6,7]. To date, 2656 human miRNAs have been identified (miRBase Release 22, [8]), many of them implicated in various diseases, such as cardiovascular, psychiatric, and neurodegenerative diseases [9]. Of note, robust evidence has shown that many miRNAs are dysregulated in cancers, acquiring functions as either oncogenes or tumor suppressors [10,11,12].

The most common form of variations present in the human genome are the single-nucleotide polymorphisms (SNPs), which occur in many genes, including miRNAs. SNPs in miRNA genes have been shown to alter either its expression or maturation, as well as its binding affinity and specificity for the target [13]. Several epidemiological studies have assessed the association of cancer susceptibility with SNPs in some miRNAs [14], with miR-146a being one of the most studied. Accumulating data suggest a link between this miRNA and cancer development; however, its specific role is controversial. An upregulated expression of miR-146a has been found in cervical cancer [15], papillary thyroid carcinoma [16], BC [17], and pancreatic cancer [17]. In contrast, miR-146a is downregulated in prostate cancer [18] and papillary thyroid carcinoma [19]. Altogether, these findings suggest that miR-146a may have diverse roles in different cancers [20].

The SNP rs2910164, located within the miR-146a precursor, involves a G>C nucleotide substitution that changes a G:U pair to a C:U pair [19]. Several studies have reported that the C allele of rs2910164:G>C increased the BC risk among European [21], Caucasian [22], and Vietnamese populations [23]. However, in North Indian populations, heterozygous GC-genotype and C-allele carriers had a reduced BC risk [24]. Moreover, association studies showed that BC patients who had at least one C allele in miR-146a were diagnosed at a younger age than patients without this variant [21]. Our group evaluated the association between rs2910164:G>C and the BC risk in a Chilean population using a case-control design. There was a significant association between the homozygous CC genotype and early-onset sporadic BC (≤50 years) [OR = 1.86, 95% CI = 1.11–3.11, *p* = 0.022]. MiR-146a-5p expression was significantly higher in BC tissues than paraneoplastic tissues [25]; nevertheless, no studies in the literature have evaluated the functional consequences of rs2910164 in the BC progress. Furthermore, the majority of reports on miRNA SNPs are based solely on association studies and, thus, provide little insight into the disease mechanism or the functional and genome-wide consequences of these mutations [26]. Therefore, the goal of the present study was to evaluate in vitro the effect of the SNP rs2910164:G>C on cell viability, apoptosis, proliferation, migration, and invasion processes involved in BC progression in luminal A and triple-negative cell lines.

## 2. Materials and Methods

### 2.1. Cell Lines

MCF-10A normal (ER-) breast epithelial cells, as well as MCF-7 luminal A (HER2-) and MDA-MB-231 triple-negative (ER-, PR-, and HER2-) BC cells were purchased from the American Type Culture Collection (Manassas, VA, USA). MCF-10A cells were cultured in DMEM-F12 (Irvine Scientific, Santa Ana, CA, USA) supplemented with 5% horse serum (Thermo Fisher Scientific, Waltham, MA, USA), 20 ng/mL EGF, 0.5 µg/mL hydrocortisone, 10 µg/mL insulin, and 100 units/mL penicillin/streptomycin. BC cells were cultured in a DMEM (Thermo Fisher Scientific, Waltham, MA, USA) supplemented with 10% fetal bovine serum (Thermo Fisher Scientific, Waltham, MA, USA), 2.5 µg/mL amphotericin B, and 100 units/mL penicillin/streptomycin (Corning, NY, USA). The cells were grown at 37 °C in a humidified atmosphere containing 5% CO_2_ and harvested for experiments after reaching a 70–80% confluence.

### 2.2. Expression Vector Design

Pre-miR-146a rs2910164-G and pre-miR-146a rs2910164-C sequences were amplified from genomic DNA extracted from homozygous rs2910164-GG and rs2910164-CC patients, with primers 5′-ATGAGTGCCAGGACTAGACC-3′ and 5′-TCTACTCTCTCCAGGTCCTCA-3′. The PCR products were agarose purified and inserted into the expression vector pcDNA3.3 TOPO-TA (Thermo Fisher Scientific, Waltham, MA, USA). The correct insertion of pre-miR-146a-G (rs2910164 G allele) and pre-miR-146a-C (rs2910164 C allele) into the expression vector was further confirmed by Sanger sequencing.

### 2.3. Cell Transfection

A mixture of 4 µL Lipofectamine 2000 (Thermo Fisher Scientific, Inc.) and 1.5 µg vector was added to the cells and left incubating for 6 h, after which the medium was replaced. The cells were transfected and then cultured for selection for 3 weeks by using 800 µg/mL G418 (Sigma Aldrich, St. Louis, MO, USA) and maintained in 400 µg/mL G418 for use in functional assays. In the case of MCF-10A, the transfection was only transient, and the cells were harvested 48 h later. MiR-146a mature levels were detected by RT–qPCR.

### 2.4. RT–qPCR Analysis of miR-146a-5p and miR-146a-3p Expression

Total cell RNA was extracted using the mirVana kit (Thermo Fisher Scientific, Waltham, MA, USA) according to the manufacturer’s protocol. MiRNA was reverse transcribed to cDNA by using the TaqMan MicroRNA Reverse Transcription kit (Applied Biosystems, Foster City, CA, USA). Mature miRNA levels were determined using specific TaqMan MicroRNA Assays (Assay ID: 000468 and 002163; Applied Biosystems, Foster City, CA, USA) in a StepOne Plus real-time PCR system (Applied Biosystems, Foster City, CA, USA). The real-time PCR was performed as follows: 95 °C for 10 min, followed by 40 cycles of 95 °C for 15 s and 60 °C for 60 s. U6 was used as a control to normalize the mature miR-146a expression. The MiRNA levels were measured based on the threshold cycle (Ct), and the relative levels were calculated using the 2^−ΔΔCt^ method [27]. All the experiments were three times performed in triplicate.

### 2.5. Proliferation Assay

A CCK-8 assay was performed according to the manufacturer’s protocol (Abcam, Cambridge, UK). Stable BC cells were seeded into 96-well plates at a density of 2 × 10^3^ cells/well in 0.1 mL of a DMEM containing 10% FBS and cultivated at 37 °C in a humidified incubator with 5% CO_2_. Following incubation for 24, 48, 72, and 96 h, each well was supplemented with 10 μL CCK-8 reagent, and the cells were incubated for an additional 2 h. The absorbance (OD) at a wavelength of 460 nm was measured in each well using a microplate reader (BioTek, Winooski, VT, USA). All the experiments were performed three times in triplicate.

### 2.6. Cell Viability Assay

The viability and chemotherapy sensitivity were determined by the MTS assay according to the manufacturer’s protocol (Promega Corporation, Madison, WI, USA). Briefly, stable BC cells were placed in 96-well plates at a density of 5 × 10^3^ cells/well in 0.1 mL DMEM. The BC cells were treated with cisplatin at concentrations of 0, 20, 40, 60, and 80 µM (Merck, CA, USA) and cultured at 37 °C in a humidified incubator for 48 h. MTS was added to each well and, the cells were incubated for 4 h at 37 °C in a humidified incubator. The absorbance was measured at 490 nm on a microplate reader (BioTek, Winooski, VT, USA), and the values were normalized using the absorbance of the untreated control cells. All the experiments were performed three time in triplicate.

### 2.7. Apoptosis Analysis

Caspase 3/7 activity was evaluated according to the manufacturer’s protocol (Promega Corporation, Madison, WI, USA). Stable BC cells were seeded into 96-well plates at a density of 1 × 10^4^ cells/well in 0.1 mL of a 10% FBS-DMEM with 0 or 100 µM cisplatin and cultured for 48 h in a humidified incubator. A caspase-Glo 3/7 reagent was added, and the cells were incubated for 3 h at room temperature with gentle agitation. The caspase 3/7 activity was measured with a luminometer: the BioTek Synergy 2 Plate Reader Multi-Mode (BioTek Instrument, Winooski, VT, USA). All the experiments were performed in triplicate.

### 2.8. Migration Assay

An 8.0 µm pore Transwell permeable support (Corning, New York, NY, USA) was used in the migration experiments. Stable MCF-7 and MDA-MB-231 BC cells were seeded into the upper chambers at a density of 1 × 10^5^ cells/well in 0.1 mL DMEM. The lower chamber contained 0.7 mL of a 10% FBS-DMEM, which was used as a chemoattractant. The cells were incubated at 37 °C in a humidified incubator for 16 h, washed with PBS, and stained at room temperature for 1 h with 1% crystal violet plus 2% methanol. Non-migrating cells in the upper chamber were removed by gentle scraping using a cotton swab, and the chambers were washed twice with ddH_2_O. The numbers of migrating cells were counted on a Nikon (Tokyo, Japan) Eclipse TS100 Inverted Routine Microscope in five random visual fields (magnification, 20×). The BC cells that were transfected with the empty vector were used as a control. The assays were conducted in three independent experiments.

### 2.9. Invasion Assays

For the cell invasion assays, the upper chambers of Transwell plates were coated with 0.1 mL of 20 µg/mL Matrigel (Merck, San Jose, CA, USA). Then, 1 × 10^5^ stable MCF-7 and MDA-MB-231 BC cells were plated in the upper chamber. The lower chamber contained 0.7 mL of a 10% FBS-DMEM. The chambers were incubated for 16 h at 37 °C, washed in PBS, and stained at room temperature for 1 h with 1% crystal violet plus 2% methanol. Five random fields were analyzed for each chamber under a Nikon Eclipse TS100 Inverted Routine Microscope at 0× magnification. The assays were conducted in three independent experiments.

### 2.10. Statistical Analysis

The statistical analysis was performed using GraphPad Prism software v10.2 (La Jolla, CA, USA). The data are presented as the mean ± SD. Comparisons of two means were performed by a *t*-test, and comparisons of multiple means were analyzed by an ANOVA. A *p* ≤ 0.05 value was considered to be a statistically significant difference.

## 3. Results

### 3.1. Pre-miR-146a-C (rs2910164:G>C) Upregulates miR-146a Expression

**Figure 1:**
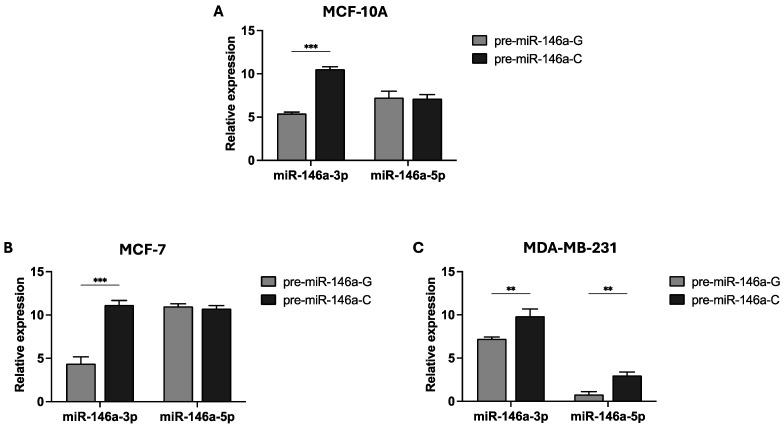
Effect of the rs2910164:G>C polymorphism on miR-146a 3p/5p expression. The rs2910164-C allele leads to the upregulation of mature miR-146a in the normal breast cell line MCF-10A (**A**) and also in the MCF-7 (**B**) and MDA-MB-231 (**C**) BC cell lines. Cells were transfected with expression vectors containing either pre-miR-146a-G or pre-miR-146a-C. Quantitative real-time PCR was used to measure the expression levels of mature miR-146a-3p and miR-146a-5p. The results are presented as log_2_ values and represent the means (±SD) of three independent experiments (* *p* < 0.05; ** *p* < 0.01; *** *p* < 0.001).

Previous findings from our group indicated that the homozygous CC genotype of rs2910164:G>C increased the BC risk in women with sporadic early-onset BC (≤50 years) (unpublished data). Therefore, we sought to investigate the effect of the pre-miR-146a rs2910164 on mature miR-146a expression in two BC cell lines: MCF-7 (GC genotype) and MDA-MB-231 (GG genotype). Pre-miR-146a produces two mature sequences: miR-146a-3p and miR-146a-5p. We constructed pre-miR-146a-G (wild-type) and pre-miR-146a-C (MAF) expression vectors, which were transfected into MCF-10A, MCF-7, and MDA-MB-231 cells. The expression levels of the two mature miRNAs were evaluated by real-time PCR. The results show that the basal level of mature miR-146a expression is low, in contrast to the levels observed in the cells transfected with the pre-miR-146a-G or pre-miR-146a-C vectors (Supplementary Table S1). As shown in Figure 1A,B, miR-146a-3p expression was significantly elevated in both the MCF-10A and MCF-7 cells transfected with pre-miR-146a-C (*p* < 0.001 and *p* < 0.001, respectively). However, no significant differences were observed in the miR-146a-5p levels between the cells transfected with either pre-miR-146a-G or pre-miR-146a-C in the normal and MCF-7 cell lines (*p* > 0.05). In the triple-negative BC cell line, transfection with pre-miR-146a-C resulted in significantly increased levels of both miR-146a-3p and miR-146a-5p compared to the G allele (*p* = 0.005 and *p* = 0.002, respectively) (Figure 1C). These findings suggest that the C allele of the SNP rs2910164 in pre-miR-146a contributes to the upregulation of mature miR-146a expression in BC cells.

### 3.2. Pre-miR-146a-C Increases Proliferation of BC Cells

**Figure 2:**
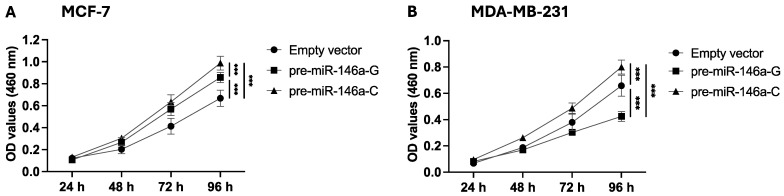
The effect of the rs2910164:G>C polymorphism on BC cell proliferation. Proliferation was evaluated using the CCK-8 assay in MCF-7 (**A**) and MDA-MB-231 (**B**) cells transfected with expression vectors carrying pre-miR-146a-G, pre-miR-146a-C, or an empty vector. The results represent the mean (±SD) of three independent experiments (* *p* < 0.05; ** *p* < 0.01; *** *p* < 0.001).

To determine the effect of rs2910164:G>C on cell proliferation, stable BC cells were analyzed by a CCK-8 assay, and the absorbance was measured at 460 nm. Cell proliferation was significantly higher at 72 and 96 h in the MCF-7 cells transfected with pre-miR-146a-C as compared to pre-miR-146a-G (*p* = 0.04 and *p* < 0.001, respectively) (Figure 2A). Figure 2B shows that the MDA-MB-231 cells transfected with pre-miR-146a-C exhibited significantly higher proliferation at 48, 72, and 96 h compared to those transfected with pre-miR-146a-G (*p* = 0.002, *p* < 0.001, and *p* < 0.001, respectively). Taken together, these findings indicate that the C allele in pre-miR-146a enhances the proliferative capacity of luminal A and triple-negative BC cells.

### 3.3. Pre-miR-146a-C Affects Cisplatin Resistance of BC Cells

**Figure 3:**
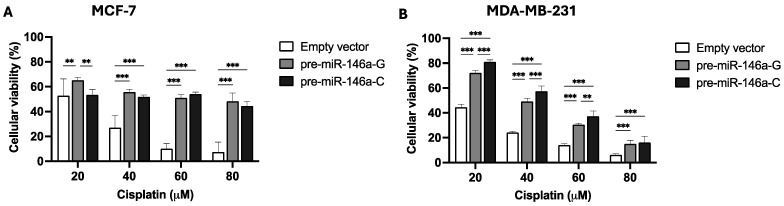
The effect of the rs2910164:G>C polymorphism on cell viability and cisplatin resistance in BC cells. Cell viability and cisplatin resistance were assessed by MTS assay after 48 h of cisplatin treatment of stable MCF-7 (**A**) and MDA-MB-231 (**B**) BC cells. The results represent the mean (±SD) of three independent experiments (* *p* < 0.05; ** *p* < 0.01; *** *p* < 0.001).

To study the effect of rs2910164:G>C on cisplatin resistance, stable BC cells were cultured for 48 h with different concentrations of the drug. An MTS assay was used to evaluate cisplatin resistance. Figure 3A shows that 20 μM of cisplatin decreased viability in the MCF-7 cells transfected with pre-miR-146a-C with respect to those transfected with pre-miR-146a-G (*p* = 0.002), but no differences were observed at 40 or 60 μM (*p* > 0.05). Nevertheless, in triple-negative BC cells, viability in the presence of cisplatin was higher in the cells transfected with pre-miR-146a-C vs. those transfected with pre-miR146a-G at 20 μM (*p* = 0.002), 40 μM (*p* = 0.024), and 60 μM (*p* = 0.026) (Figure 3B). In conclusion, these results indicate that the overexpression of mature miR-146a decreases cell viability and cisplatin resistance in MCF-7 cells. Nevertheless, significantly increased cell viability and cisplatin resistance were observed in the triple-negative cells transfected with pre-miR-146a-C as compared to those transfected with pre-miR146a-G. Therefore, the effect of cisplatin on viability and cell drug resistance appears to be subtype-specific.

### 3.4. Pre-miR-146a-C Expression Decreases Cisplatin-Induced Apoptosis of BC Cells

**Figure 4:**
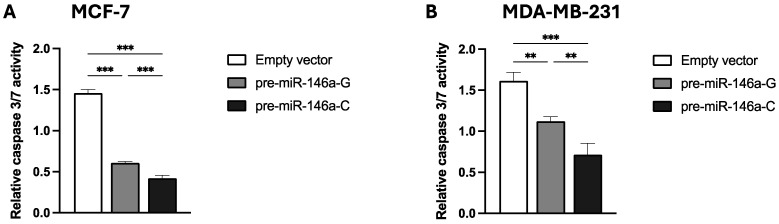
The effect of the rs2910164:G>C polymorphism on cisplatin-induced apoptosis in BC cell lines. Caspase 3/7 activity was measured to evaluate apoptosis in stable MCF-7 (**A**) and MDA-MB-231 (**B**) BC cells transfected with vectors containing pre-miR-146a-G, pre-miR-146a-C, or empty vector. The results represent the mean (±SD) of three independent experiments (* *p* < 0.05; ** *p* < 0.01; *** *p* < 0.001).

To evaluate the impact of the rs2910164:G>C polymorphism on cisplatin-induced apoptosis, the stable BC cells transfected with pre-miR-146a-G, pre-miR-146a-C, or empty vector were exposed to 100 μM cisplatin. The apoptosis activity was quantified by assessing caspase 3/7 enzymatic activity. Figure 4A,B show that the cisplatin-induced apoptosis at 48 h was significantly lower in both the MCF-7 and MDA-MB-231 BC cells transfected with pre-miR-146a-C vs. those transfected with pre-miR-146a-G (*p* < 0.001 and *p* = 0.003, respectively) or an empty vector (*p* < 0.001 and *p* < 0.001, respectively). These results indicate that miR-146a upregulation, attributable to the presence of pre-miR-146a-C, decreases cisplatin-induced apoptosis. The results are in agreement with our previous finding indicating that rs2910164-C is a risk-heightening allele.

### 3.5. Pre-miR-146a-C Increases Migration and Invasion of BC Cells

**Figure 5:**
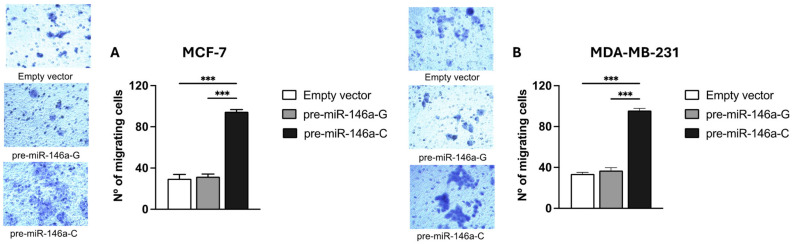
The effect of the rs2910164:G>C polymorphism on migration in BC cell lines. Migration was assessed using Transwell chamber assay in stable MCF-7 (**A**) and MDA-MB-231 (**B**) cells transfected with expression vectors containing pre-miR-146a-G, pre-miR-146a-C, or empty vector using Transwell chambers. The numbers of migrating cells were quantified by counting cells under light microscopy at 20× magnification in 5 randomly selected fields. The results represent the mean (±SD) of three independent experiments (* *p* < 0.05; ** *p* < 0.01; *** *p* < 0.001).

**Figure 6:**
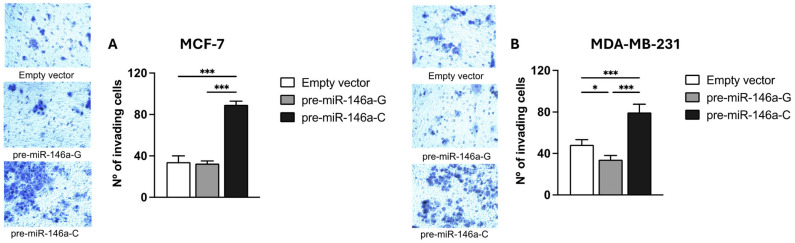
The effect of the rs2910164:G>C polymorphism on invasion in BC cell lines. Cell invasion was evaluated by using Matrigel-coated Transwell plates in MCF-7 (**A**) and MDA-MB-231 (**B**) cells transfected with pre-miR-146a-G, pre-miR-146a-C, or empty vectors. Invading cells were quantified by light microscopy at 20× magnification in 5 randomly selected fields. The results represent the mean (±SD) of three independent experiments (* *p* < 0.05; ** *p* < 0.01; *** *p* < 0.001).

BC cell migration and invasion were evaluated using Transwell chambers with or without a Matrigel coating, respectively. Both the MCF-7 and MDA-MB-231 cells transfected with the pre-miR-146a-C vector exhibited significantly higher migratory activity compared to those transfected with pre-miR-146a-G (*p* < 0.001 and *p* < 0.001, respectively) (Figure 5A,B). Similarly, a significant increase in the invasive capacity was observed in the same cell lines transfected with pre-miR-146a-C vs. pre-miR-146a-G (*p* < 0.001 and *p* < 0.001, respectively) (Figure 6A,B). Furthermore, the cells transfected with pre-miR-146a-C showed significantly elevated levels of both migration and invasion compared to those transfected with the empty vector (*p* < 0.001). These findings indicate that the overexpression of miR-146a, driven by the rs2910164 C allele in pre-miR-146a, enhances migration and invasion in luminal A and triple-negative BC cells.

## 4. Discussion

MiRNAs play a major role in regulating gene expression. Deregulated miRNA expression and abnormal miRNA function are associated with various diseases, and the role of miRNAs in cancer is of particular recent interest. Variations in primary, precursor, or mature miRNA can influence pri-miRNA transcription, the processing of precursors to mature miRNAs, as well as miRNA-mRNA interactions, all of which are potential factors in cancer development [11]. miR-146a is one of the most extensively studied miRNAs. Its dysregulation has been associated with cancer development, and it has been implicated in various processes, such as cell metastasis, proliferation, migration, invasion, apoptosis, and survival [28]. miR-146a is also associated with multiple malignancies, including breast, pancreatic, and gastric cancers [29]. However, while many publications suggest a role for miR-146a in BC, the results regarding the mechanistic functions of this miRNA are conflicting [30].

There is a consensus that SNPs are related with the progression of several human cancers [31,32]. The SNP rs2910164 is located within the miR-146a precursor and involves a G>C nucleotide substitution changing a G:U pair to a C:U pair [19]. It is reported that the C allele of rs2910164 increases the BC risk in European [21] and Caucasian [22] populations. A study developed in a Vietnamese population also revealed an increased risk for CG-genotype carriers [23]. However, the GC-genotype and C-allele carriers showed a reduced BC risk in an Indian population [24]. On the other hand, Shen et al. [33] studied the association between the SNP miR-146a rs2910164 and the age at diagnosis in 42 patients with familial BC, finding that patients carrying the C allele were diagnosed at an earlier age than GG homozygotes. These results were confirmed by Pastrello et al. [34], who reported that patients with the GC or CC genotype developed tumors at an earlier age than individuals with the GG genotype, suggesting that rs2910164-C may relate with an increased risk of BC development at an early age. Furthermore, the literature shows that the SNP rs2910164:G>C can contribute significantly to BC pathogenesis, since the targets of miR-146a include *BRCA1* and *BRCA2* [33,35], genes that are key in BC and breast–ovarian syndrome. Association studies have shown that BC patients who had at least one C allele in miR-146a were diagnosed at an earlier age than patients without this variant [33]. Our results have also demonstrated a significant association between the homozygous CC genotype and early-onset sporadic BC (≤50 years) in a Chilean population, which is an admixture of Caucasian and Asian groups [36]. However, the variant rs2910164:G>C has not been widely investigated in relation to its functional impact on cancer cells. Considering the above-described results, in this study, we performed an in vitro evaluation of the role of the SNP rs2910164:G>C in processes related to BC progression, using luminal A and triple-negative cell lines.

We evaluated the expression of miR-146a 5p and 3p strands in the normal breast cell line MCF-10A and in the BC cell lines MCF-7 and MDA-MB-231 transfected with pre-miR-146a-G or pre-mir-146a-C expression vectors. Our results showed that the presence of the rs2910164-C allele in the pre-miR-146a gene led to an increased expression of the mature miR-146a-3p strand in the normal as well as in the BC luminal A and triple-negative cell lines, while the expression of the mature miR-146a-5p strand was augmented only in the triple-negative BC cells. As the SNP rs2910164:G>C is located in an internal loop of the pre-miR-146a—a part of the miR-146a-3p seed region - this might impact the secondary structure of the miR-146a precursor. In fact, it has been proposed that a cytosine would create two internal loops around the Dicer cleavage site, whereas a guanine would create only one inner loop [37]. This suggests that the presence of the C allele leads to the greater production of mature miR-146a compared to the G allele, which aligns with our results and those reported by Shen et al. [33]. Additionally, Jazdzewski et al. [19] identified an association between the rs2910164 polymorphism and an increased risk of developing papillary thyroid cancer, where this SNP was shown to interfere with the processing of miR-146a [19]. In individuals homozygous for this variant, two mature miRNAs are generated—miR-146a from the leading strand, and either miR-146a-G or miR-146a-C from the passenger strand. In contrast, heterozygous individuals produce three mature forms: miR-146a-5p, along with both miR-146a-3p-G and miR-146a-3p-C. Thus, these three miRNAs, and consequently the regulation of their multiple target genes, may explain the risk of papillary thyroid cancer observed in heterozygous patients [19]. Accordingly, a similar situation could occur in BC but also in other cancers in which the miR-146a overexpression has been reported, such as cervical [15], papillary thyroid [16], pancreatic [17], bladder [38], and gastric [20] cancer.

The rs2910164:G>C in pre-miR-146a also had effects on BC cell proliferation. Our results showed that the presence of rs2910164-C led to increased BC cell proliferation in the MCF-7 and MDA-MB-231 cell lines. It is probable that the upregulated mature miR-146a expression, as a consequence of the presence of the rs2910164 C allele, enhances BC cell proliferation. Thus, in our knowledge, our findings are the first to suggest a functional consequence of rs2910164:G>C in luminal A and triple-negative BC. 

Cisplatin is commonly used in the treatment of several solid tumors, including those affecting the testicles, ovaries, bladder, lungs, cervix, head and neck region, and stomach [39,40,41]. However, many cancers display intrinsic or acquired resistance to cisplatin and other drugs [42,43]. Recent studies show that miRNAs might regulate chemotherapy resistance [44]. For instance, Yang et al. [45] described that miR-214 promotes survival and cisplatin resistance by targeting the PI3K/Akt pathway in ovarian cancer. Jiang et al. [44] reported that miR-146a modulated cisplatin resistance via the NF-κB pathway in non-small cell lung cancer. Hu et al. [46] confirmed that miR-146a overexpression promoted cervical cancer cell viability. In triple-negative BC cells, our findings indicated that miR-146a overexpression, as a consequence of the rs2910164 C allele in pre-miR-146a, led to increased survival after cisplatin exposure. Several publications have also found that miR-146a is overexpressed in triple-negative BC [30]. However, in MCF-7 cells with the rs2910164 C allele in pre-miR-146a, we only observed decreased survival at 20 μM cisplatin. At greater cisplatin concentrations, the drug sensitivity was similar in the presence of rs2910164-G or rs2910164-C. Therefore, the SNP rs2910164 did not promote cisplatin resistance in the luminal A BC cell line. It seems that the effect of cisplatin on cell viability and drug resistance is subtype-specific.

Apoptosis is a major barrier that cancer must evade in order to develop. Several studies have reported that miR-146a plays a significant role in apoptosis [29]. Liu et al. [47] reported that increased miR-146a-5p expression reduced apoptosis in non-small cell lung cancer, while decreased expression resulted in increased apoptosis. In cervical cancer cells, miR-146a overexpression promoted cell growth and reduced apoptosis [48]. In gastric cancer cells, miR-146a overexpression improved cell proliferation and inhibited apoptosis, as well as downregulated *SMAD4* gene expression [20]. According to our findings, elevated miR-146a levels, stemming from the rs2910164-C allele, inhibited apoptosis in both MCF-7 and MDA-MB-231 BC cells, consistent with observations in other malignancies. *SMAD4*, a known tumor suppressor, plays a central role as a mediator in the TGF-β signaling pathway. Recent studies have suggested that understanding the role of the TGF-β pathway and SMAD proteins in BC may significantly inform its diagnosis, treatment, and prognosis [49,50]. Several studies have indicated that the loss of SMAD4 is frequent in multiple cancers, such as lung, colon, breast, and gastric cancers [51,52,53,54]. Considering that *SMAD4* is a target of miR-146a, it is possible that miR-146a overexpression, as a consequence of the rs2910164 C allele, inhibits apoptosis via the downregulation of SMAD4, as occurs in gastric cancer.

Metastasis is the leading cause of mortality in most cancers. Cell migration and invasion are the main manifestations of tumor biology and are critical components of metastasis [55]. Several studies have demonstrated that miR-146a is involved in metastasis in cancer [56,57]. In non-small cell lung cancer, miR-146a-5p overexpression was found to promote proliferation and migration via the direct suppression of TRAF6 [47], and in oral squamous cell carcinoma, greater miRNA-146a expression was observed to enhance cell migration and invasion [58]. Similarly, Wang et al. [59] demonstrated that miR-146a promotes proliferation, cell cycle progression, migration, and invasion in liver cell lines, and in follicular thyroid carcinoma, increased miR-146a levels promoted cell proliferation, migration, and invasion. In melanoma, miR-146a has been shown to promote cell migration and invasion [60]. This study showed that the presence of the rs2910164 C allele led to the enhanced migration and invasion of BC cells, likely due to miR-146a overexpression. Multiple studies support that miR-146a influences the expression of genes involved in cancer progression and metastasis by modulating key signaling pathways, including TNFα/TRAF6, IFNγ/STAT, GPCR, PI3K-AKT-mTOR, and TGFβ/SMAD [28,61]. In follicular thyroid carcinoma, miR-146a promotes cell migration and invasion by downregulating *ST8SIA4* mRNA and affecting the PI3K-AKT-mTOR axis [62]. Additionally, miR-146a promotes NF-kB signaling in oral, cervical, breast, and prostate cancers through the targeting of *IRAK1* and *TRAF6* [30,46,61], and it enhances cell growth and proliferative signaling in several cancers—including breast, liver, lung, prostate, and gastric—by targeting *EGFR* [30]. In oral cancer, miR-146a targets the *HTT* gene; in liver cancer, the *FLAT* mRNA; and in melanoma, SMAD4, regulating cell migration and invasion [59,60,63]. Our results show that miR-146a upregulation in the presence of the pre-miR-146a rs2910164 C allele enhanced migration and invasion in luminal A and triple-negative BC cells, which could occur through the regulation of the TGFβ/SMAD pathway or the targeting of *HTT* and/or *SMAD4*. MiR-146 targets *HTT*, and miR-146a overexpression reduces HTT expression, which is related to cancer metastasis and lower patient survival in BC [63].

## 5. Conclusions

Mature miR-146a-3p expression is upregulated in luminal A and triple-negative cell lines due to the presence of the rs2910164-C allele in pre-miR-146a. Pre-miR-146a-C increases proliferation, migration, and invasion in luminal A and triple-negative BC cells and decreases apoptosis. The effect of pre-miR-146a-C on cisplatin resistance is subtype-specific. All the in vitro results confirm that rs2910164-C is a risk-heightening allele in BC.

## Data Availability

The original contributions presented in this study are included in the article/Supplementary Materials. Further inquiries can be directed to the corresponding author(s).

## Supplementary Materials

The following supporting information can be downloaded at https://www.mdpi.com/article/10.3390/cells14080612/s1. Table S1: Expression of miR-146a in BC Cells.
