# Supplementary material for: The miR-146a Single Nucleotide Polymorphism rs2910164 Promotes Proliferation, Chemoresistance, Migration, Invasion, and Apoptosis Suppression in Breast Cancer Cells
Source: Cells. 2025 Apr 18;14(8):612. doi: 10.3390/cells14080612 (PMC12025401; doi:10.3390/cells14080612)
Supplement: Supplementary file 1 [file cells-14-00612-s001.zip › cells-3476713-supplementary.pdf]

Table S1: Expression of miR-146a in BC Cells. Level of mature miR-146a expression in MCF-7 (A) and MDA-MB-231 (B) cells transfected with the empty vector (basal level), pre-miR-146a-G, or pre-miR-146a-C

A.

MCF-7	Relative expression ($2^{-\Delta\Delta Ct}$)	log ($2^{-\Delta\Delta Ct}$)	log_2 ($2^{-\Delta\Delta Ct}$)
VV	20.1213	1.3036	4.3306
G	6304.1370	3.7996	12.6220
C	12358.1116	4.0919	13.5931

B.

MDA-MB-231	Relative expression ($2^{-\Delta\Delta Ct}$)	log ($2^{-\Delta\Delta Ct}$)	log_2 ($2^{-\Delta\Delta Ct}$)
VV	8.6321	0.9361	3.1097
G	461.4942	2.6641	8.8501
C	3105.5991	3.4921	11.6006

.
